# Corticosteroids for all adult patients with community-acquired pneumonia?

**DOI:** 10.15172/pneu.2015.6/690

**Published:** 2015-12-01

**Authors:** Simone M. C. Spoorenberg, Stefan M. T. Vestjens, Werner C. Albrich, Ger T. Rijkers

**Affiliations:** 16 Department of Internal Medicine, St. Antonius Hospital, Nieuwegein, The Netherlands; 26 Division of Infectious Diseases and Hospital Epidemiology, Kantonsspital, St. Gallen, Switzerland; 36 Department of Medical Microbiology and Immunology, St. Antonius Hospital, Nieuwegein, The Netherlands; 46 Department of Science, University College Roosevelt, Lange Noordstraat 1 4331CB, Middelburg, The Netherlands

**Keywords:** community-acquired pneumonia, corticosteroids, immunomodulation, adult, randomised-controlled trial

Corticosteroid therapy as adjunctive treatment in community-acquired pneumonia (CAP) is a promising but controversial subject. The potentially beneficial effect of corticosteroids is based on the ability of steroids to dampen an excessive inflammatory response that often occurs in patients with CAP. This excessive inflammatory response can cause damage to the lungs and other organs, and is associated with poor outcome [1].

Over the years, there have been several observational studies evaluating the use of corticosteroids in patients with CAP, although these studies produced conflicting results. In a Spanish single-centre cohort study of patients with severe CAP (Pneumonia Severity Index [PSI] IV/V), patients who received corticosteroids had a significantly lower 30-day mortality (odds ratio [OR] 0.29) than patients without corticosteroids [2]. In contrast, in a smaller retrospective cohort study performed in 2 intensive care units (ICUs) in Brazil and Portugal, steroids were not associated with a reduction in mortality [3]. A propensity score-matched study of a Japanese administrative database, which included 6,925 patients with severe CAP who required mechanical ventilation, found no mortality benefit in patients without shock, but did find a significant reduction in 28-day mortality in patients with septic shock [4]. A single-centre study evaluating 3,257 patients with CAP of any severity in Barcelona from 1997 to 2008 found that corticosteroids were given to patients who tended to be sicker. Nevertheless, no mortality difference was identified between those with and those without corticosteroids [5]. The main and very relevant limitation of all these studies lies in their observational nature, with major imbalances in baseline characteristics of patients with and without corticosteroids. Due to these methodological limitations, randomised trials are clearly better suited to provide information about the real value of corticosteroids in CAP.

Sixty years ago, the first quasi-randomised clinical trial (RCT) on corticosteroids in CAP was performed by Wagner and colleagues [6]. They administered oral hydrocortisone (560 mg over 5 consecutive days) to 52 patients with pneumococcal pneumonia, and compared clinical parameters with 61 patients who received standard care only [6]. They noticed a more rapid defervescence and faster improvement in symptoms in the hydrocortisone group, although symptoms reappeared when hydrocortisone was withdrawn. At that time, reviewers did not ask for any statistical analysis when accepting a paper for publication, so we are not informed about the significance of their findings. It was concluded that hydrocortisone was a safe and promising medicine for further exploration. Still, it took almost 20 years until the next RCT on corticosteroids in CAP was published. This trial—not even primarily designed to explore the corticosteroid effect but merely to investigate ampicillin dose—showed a trend for faster defervescence with prednisolone (5 mg every 6 hours given for 7 days) but no difference in mortality [7]. Another 20 years later in 1993, Marik and colleagues [8] investigated the effect of a single hydrocortisone bolus of 10 mg/kg on tumour necrosis factor (TNF)-α values in patients admitted to the ICU with severe CAP in South Africa. No differences in TNF-α levels or in mortality were detected in that study. In 2004, the first well-designed RCT with a longer course of corticosteroids was performed. In this trial, patients who were admitted to the ICU with severe pneumonia received a 200 mg hydrocortisone bolus, followed by hydrocortisone (10 mg/h for 7 days) [9]. After an interim analysis performed on 46 patients, the trial was stopped due to a significant difference in improvement in the primary endpoint (partial pressure arterial oxygen [PaO_2_]/fractional inspired oxygen [FiO_2_] ratio) 8 days after inclusion and a lower in-hospital mortality rate (0 vs. 30%), both favouring the hydrocortisone group. Since then, 4 RCTs that only included patients with severe CAP have been published, all describing positive results regarding the corticosteroid group [10–13]. Faster improvement in PaO_2_/FiO_2_, a reduction in delayed septic shock, and shorter duration of mechanical ventilation were found in the corticosteroid groups. None of the studies found a difference in mortality (either short-term or long-term). The study by Torres et al [10] was notable in that it required almost 8 years in 3 centres to recruit 113 patients, who had some residual imbalances in baseline characteristics. Methylprednisolone was given within 36 hours of enrolment. While no effect was seen in early treatment failures (i.e. within 72 hours), late failures were reduced in patients who received methylprednisolone.

Results of RCTs that have included patients with all ranges of CAP severity are variable and sometimes conflicting. Snijders and colleagues [14] found a faster defervescence but not a faster time to recovery in patients receiving 40 mg of prednisone for 7 consecutive days, and also found a higher risk of late failures after initial improvement. Since this latter finding has not been reproduced in subsequent studies, one proposed explanation for this was a random effect. In an open-label RCT, Mikami et al. [15] did not find an effect on duration of hospital stay (their primary endpoint), but did detect a faster time to recovery and shorter duration of intravenous antibiotic therapy. On the contrary, Blum et al. [16] and Meijvis et al. [17] did show a significant faster time to clinical stability and a shorter length of stay in more recent Swiss and Dutch studies, respectively. Furthermore, Nafae and colleagues [18] showed improvement in PaO_2_/FiO_2_ ratio and a reduction in duration of mechanical ventilation. Again, no differences in mortality were found. In general, throughout these RCTs there was surprisingly little evidence of adverse events with the notable exception of hyperglycaemic episodes, some of which required insulin treatment.

Due to the different types of corticosteroids used in the various studies, the variation in dosages and dosing regimens, differences of entry criteria and thus study populations, the variability of the antibiotic regimens including immunomodulatory antibiotic classes (particularly macrolides and maybe fluoroquinolones), and the heterogeneity in outcome measures, the available studies are difficult to compare. With generally improving treatment standards and quality of care in developed countries, it becomes increasingly difficult to show mortality benefits from new interventions. Not surprisingly, the main benefit seems to relate to various other endpoints, particularly time to stability, length of stay, and possibly duration of intravenous antibiotics [16,19]; all advantageous from economic or public health points of view. In addition, a meta-analysis published prior to the most recent and largest RCTs did find a mortality benefit for the subgroup of patients with severe CAP, but not in all patients with CAP [20].

Therefore, future studies will need to define specific subgroups that benefit more from corticosteroid therapy. For example, patients with severe sepsis appear to benefit most from corticosteroid therapy, considering the results of the 4 severe-CAP trials [10–13] were all positive. Furthermore, in the study of Mikami et al. [15], selecting patients with a moderate to severe CAP (according to the American Thoracic Society criteria), the corticosteroid group did show a shorter duration of intravenous antibiotic use and a faster stabilisation of vital signs compared to the placebo group. However, in the study of Blum et al. [16], both patients with mild to moderate pneumonia (PSI I–III) and patients with severe pneumonia (PSI IV/V), benefitted similarly from corticosteroids with no effect modification due to PSI. Interestingly, an improved time to stability remained in all reported subgroup analyses in this study. Rather than the PSI score, maybe we need to use alternative classification schemes (such as a combination of inflammatory mediators) to select and identify the patients who would benefit most from the immunomodulatory effects of corticosteroids. Torres et al. [10] included only patients with a C-reactive protein >150 mg/dL and found less treatment failure in the group who received methylprednisolone for 5 consecutive days. Another possible subgroup is patients with relative adrenal insufficiency (RAI). These patients might benefit more from the glucocorticoid effect of the corticosteroid therapy, compared to patients without RAI [21]. It should be admitted, however, that in the study of Mikami et al. [15] no difference in outcome between patients with and without RAI was found.

CAP caused by certain pathogens may have more advantage from corticosteroid therapy, considering the faster decline in cytokines in the atypical pathogens *Mycoplasma pneumoniae, Coxiella burnetii, Legionella pneumophila* and *Chlamydia* species compared to *Streptococcus pneumoniae* on a 4-day course of dexamethasone [22]. Of note, the study of Snijders and colleagues [14] had a significantly higher failure rate on day 30 for patients with pneumococcal pneumonia. Other observational data documented a higher risk of detrimental outcomes including mortality in severely ill patients with pandemic 2009 influenza A (H1N1) who received corticosteroids [23,24]. Patients with pre-existent cardiovascular disease might be a target group if corticosteroids can reduce the increased short-term and long-term cardiovascular risk after pneumonia [25]. One of the hypotheses is that the pneumonia episode leads to pro-inflammatory changes of pre-existing atherosclerotic lesions. This makes the lesions more vulnerable to plaque rupture and therefore susceptible to causing a cardiovascular incident. Additionally, high interleukin (IL)-6 levels at time of discharge are associated with increased 1-year cardiovascular mortality [26]. Corticosteroids can reduce IL-6 levels and might prevent the atherosclerotic lesion changes, thereby possibly reducing cardiovascular risk after pneumonia.

Until now, corticosteroids do seem to have a beneficial effect for those patients hospitalised with CAP who fulfil the inclusion and exclusion criteria of the recent RCTs. However, since hyperglycaemia is an often seen side effect, future research needs to be focused on the question if there are specific CAP subgroups that would have substantial advantage of corticosteroid therapy. The Santeon-CAP trial (ClinicalTrials.gov registry: NCT01743755), stratifying patients according to PSI severity, is currently underway and could provide additional criteria to determine which patients would benefit most from steroids. Other large trials are needed to further investigate advantages in other subgroups, such as patients with RAI, certain pathogens (including influenza virus type A and methicillin-resistant *Staphylococcus aureus*), or specific comorbidities, and their added benefit in regimens including or excluding macrolide antibiotics.

Finally, apart from short-term effects (duration of hospital stay, 30-day mortality, quality of life) also long-term mortality should be considered when deciding on the use of steroids for adjunctive treatment of CAP.
